# Different Modes of Performing Lithotrity in the English Hospitals

**Published:** 1859-11

**Authors:** 


*Different Modes of Performing Lithotrity in the English Hospitals.*

A large majority of English surgeons employ the ordinary, lateral method of lithotomy, on a curved staff. There has been, however, a considerable disposition to endeavor to improve on it of late years. The median plan, so strongly recommended by Mr. Allarton, has been tried by not a few London surgeons, and amongst provincial ones has found a warm advocate in Mr. Teale, of Leeds. At the London Hospital it was first adopted by Mr. Ward, about two years ago, and since then has been employed by his colleagues, Mr. Critchett and Mr. Gowlland, each in a single instance. All the three patients were children, all recovered well, and in all it was considered that much less than the usual amount of bleeding took place. At Guy's Hospital, Mr. Cock has performed median lithotomy several times; and Mr. Erichsen has done the same at University College Hospital; both surgeons being, we believe, well satisfied with its results. On all hands it is considered to be best adapted for children and for small stones. At St. Bartholomews, Mr. Lloyd still continues to operate in all cases by his recto-urethral (median) method, which we described in detail when he first adopted it in 1853. He informs us that he has not yet lost a case after it, and considers it decidedly preferable to the lateral operation. His colleagues, however, without exception, we believe, always employ the latter. At the Metropolitan Free, Mr. Hutchinson always employs his rectangular catheter-staff, and considers that he obtains great advantage from it. The same instrument has been employed at King's College by Mr. Lee; but it is not, as far as we observe, in use at any other hospitals. In a recent instance in which the calculus was of large size, Mr. Hutchinson injected the bladder with oil instead of water, in the hope of facilitating the dilatation of the parts.

With regard to the median operation, as advised by Mr. Allarton, it is universally admitted to be adapted only for small calculi. Now, Mr. Lloyd's experience during the last few years has quite proved that, when the anterior commissure of the sphincter ani is cut clean through from the perineal wound, there is no danger of the parts not healing. Might it not be well, therefore, to adopt this measure whenever, after the usual median incisions, the stone has been reached and found too large for removal? Mr. Lloyd's operation gives abundance of room.—*Med. Times and Gaz.*, July 9, 1859.

